# Dysbiotic Bacterial and Fungal Communities Not Restricted to Clinically Affected Skin Sites in Dandruff

**DOI:** 10.3389/fcimb.2016.00157

**Published:** 2016-11-17

**Authors:** Renan C. Soares, Pedro H. Camargo-Penna, Vanessa C. S. de Moraes, Rodrigo De Vecchi, Cécile Clavaud, Lionel Breton, Antonio S. K. Braz, Luciana C. Paulino

**Affiliations:** ^1^Centro de Ciências Naturais e Humanas, Universidade Federal do ABCSanto André, Brazil; ^2^L'Oréal, Research and InnovationRio de Janeiro, Brazil; ^3^L'Oréal, Research and InnovationAulnay-sous-Bois, France

**Keywords:** dandruff, skin, microbiota, *Malassezia*, dysbiosis, next generation sequencing

## Abstract

Dandruff is a prevalent chronic inflammatory skin condition of the scalp that has been associated with *Malassezia* yeasts. However, the microbial role has not been elucidated yet, and the etiology of the disorder remains poorly understood. Using high-throughput 16S rDNA and ITS1 sequencing, we characterized cutaneous bacterial and fungal microbiotas from healthy and dandruff subjects, comparing scalp and forehead (lesional and non-lesional skin sites). Bacterial and fungal communities from dandruff analyzed at genus level differed in comparison with healthy ones, presenting higher diversity and greater intragroup variation. The microbial shift was observed also in non-lesional sites from dandruff subjects, suggesting that dandruff is related to a systemic process that is not restricted to the site exhibiting clinical symptoms. In contrast, *Malassezia* microbiota analyzed at species level did not differ according to health status. A 2-step OTU assignment using combined databases substantially increased fungal assigned sequences, and revealed the presence of highly prevalent uncharacterized *Malassezia* organisms (>37% of the reads). Although clinical symptoms of dandruff manifest locally, microbial dysbiosis beyond clinically affected skin sites suggests that subjects undergo systemic alterations, which could be considered for redefining therapeutic approaches.

## Introduction

Human skin is a complex ecosystem inhabited by a variety of microorganisms, including bacteria, fungi, archaea, and viruses (Kong, 2011). Our knowledge of the skin microbiome has increased substantially in recent years, driven by advances in sequencing technologies and in bioinformatics (Tomic-Canic et al., 2014). These approaches have shown interpersonal, topographical, and temporal variations of microbial communities (Grice et al., 2009; Caporaso et al., 2011; Findley et al., 2013; Oh et al., 2014, 2016).

Skin microbiome has positive impact on several aspects of human health, such as immune response modulation and protection against pathogens (Wanke et al., 2011; Naik et al., 2015). Microorganisms interact with host keratinocytes and innate immune system, stimulating the secretion of antimicrobial peptides, free fatty acids, cytokines and chemokines, which might lead to adaptive immune responses (Gallo and Nakatsuji, 2011; Fyhrquist et al., 2016).

Microbial communities from skin have also been associated with the development of skin disorders (Grice, 2014). Dandruff is one of the most common skin conditions, affecting approximately half of adult population worldwide (Piérard-Franchimont et al., 2006a). This inflammatory chronic disorder is related to skin barrier disruption, epidermal cellular proliferation and differentiation, as well as shifts in gene expression patterns, and in cytokine and lipid production (Kerr et al., 2011; Mills et al., 2012; Bonnist et al., 2014). It is characterized by erythema, itching and scaling on scalp (Schwartz et al., 2013), and is also related to alopecia (Piérard-Franchimont et al., 2006b). Dandruff also has social and psychological impact, affecting self-esteem, and confidence (Manuel and Ranganathan, 2011). It has been frequently associated with yeasts from *Malassezia* genus, which are also members of the healthy cutaneous microbiome (Saunders et al., 2012). However, the role *Malassezia* organisms play in the development of the symptoms has not been elucidated and the etiology of dandruff remains poorly understood.

We used next-generation sequencing (NGS) to analyze bacterial and fungal microbiota associated with skin from healthy and dandruff subjects. The comparison between lesional and non-lesional skin sites from dandruff subjects provided new perspectives for the understanding of this skin disorder, establishing steps toward a broader view of dandruff etiology and the role of the microbiome in the symptom development.

## Materials and methods

### Subjects and sample collection

The research protocol was approved by the UFABC Institutional Review Board (Protocol 732.172) and was conducted according to the principles expressed in the World Medical Association Declaration of Helsinki. All subjects provided written informed consent prior to any study-related procedures.

Thirteen patients with dandruff and 11 healthy subjects were enrolled, with the collaboration of “Instituto Superior de Medicina e Dermatologia—ISMD” (São Paulo, SP, Brazil). Volunteers were individuals from both genders, ages between 18 and 61 years old (Supplementary Table 1). All participants provided information regarding health status, medical history, and daily habits. Volunteers were non-smokers, did not have cutaneous diseases except dandruff, did not receive antibiotics or systemic antifungals 1 month prior to sampling, and did not use anti-dandruff shampoos and chemical products (dyeing, bleaching, permanent waving, straightening etc.) on scalp and hair at least 2 weeks prior to sampling.

Volunteers were asked to use a neutral shampoo 3 times a week during 2 weeks prior to sampling to standardize the scalp condition. They were advised not to wash their scalp 2 days before the sampling procedure, and not to use other hair products on the scalp (Clavaud et al., 2013).

Dandruff severity was measured as previously described (Clavaud et al., 2013). Scalp was divided into eight sections and dandruff scores ranging from 0 to 5 were assigned to each area by comparison with reference pictures. The values were averaged to obtain the final score. Samples from scalp (vertex of the head) and forehead (central area) were obtained using sterile cotton swabs soaked in a solution containing 0.15M NaCl and 0.1% Tween 20, as previously described (Paulino et al., 2006). Forehead from dandruff subjects did not show any sign of desquamation or inflammation. Swabs were placed in microcentrifuge tubes, transported in dry ice, and stored at −80°C. Cotton swabs with no skin contact submitted to the same procedures were used as negative controls.

### DNA extraction, PCR amplifications, and high-throughput sequencing

Total genomic DNA was extracted from the swabs by using Power Soil DNA Isolation Kit (MOBIO Laboratories Inc., Carlsbad, CA, USA) according to the manufacturer's instructions. The head of each swab was cut from the handle and placed into a tube provided by the kit, which contained beads to efficiently disrupt cell walls.

Primers 520F (Claesson et al., 2009) (5′-AYTGGGYDTAAAGNG-3′) and 907R (Lane et al., 1985) (5′-CCGTCAATTCMTTTRA-3′) were used to amplify by PCR a fragment containing the V4 hypervariable region of the 16S rRNA gene from bacteria. For fungi 18S-F/5.8S-1R ITS1 primers were used (Findley et al., 2013). Sequencing was performed using MiSeq Illumina Platform with Illumina paired-end MiSeq Reagent Kit v2 (2 × 250) following Illumina's standard protocol. Only reads obtained with forward primers were considered in the analyses as they showed higher sequencing quality. Single reads were submitted to size filtering using Seqyclean software (https://bitbucket.org/izhbannikov/seqyclean). Sequences shorter than 200 bp were excluded from the analyses. Quality filtering was done using Phred quality score (≥20; Ewing et al., 1998). Size and quality filtering were performed using Qiime pipeline (Caporaso et al., 2010).

The dataset has been deposited in the EBI Metagenomics database (project number PRJEB16723).

### Taxonomic assignment

Bacterial taxonomic assignment was performed considering 97% identity and 95% coverage by comparing the sequences with Greengenes database v 13_8 (http://greengenes.lbl.gov/) using Uclust software (Edgar, 2010), implemented by Qiime. Fungal community analyses were done at genus level through BLAST (Altschul et al., 1990) using QIIME against a database of ITS1 reference sequences manually curated from UNITE database v 2015-03-02 (https://unite.ut.ee/), with 70% similarity threshold. Singletons were removed from the analysis. A database of *Malassezia* sequences were constructed using all the sequences from *Malassezia* organisms in the UNITE database that were manually curated (reference sequences—Kõljalg et al., 2013), combined with Genbank sequences presenting ≥97% similarity and ≥95% coverage in relation to those selected from the Unite database. Analyses at species level for *Malassezia* organisms were performed considering 97% similarity threshold and ≥95% coverage. Fungal unassigned sequences were clustered using CD-HIT (Li and Godzik, 2006; 97% similarity threshold), and clusters with relative abundance ≥1% in at least one sample were compared through BLAST against the complete Genbank database. This step of analysis also considered three *Malassezia* species described recently: *M*. *brasiliensis, M. pscittasci* (Cabañes et al., 2016) and *M. arunalokei* (Honnavar et al., 2016), which were not included in the *Malassezia* sequence database built with UNITE and Genbank.

### Statistical analysis

Non-metrical Multidimensional Scaling (nmMDS) using Bray-Curtis similarity distances (Bray and Curtis, 1957) based on Log (X+1) transformed data were performed to assess the relationships between bacterial communities from different samples. Analysis of Similarities (ANOSIM; Clarke, 1993) was applied to verify differences based on body site and health condition. ANOSIM global R value ranges from 1 to −1 (R ~ 0 indicates the same level of variation within and between groups). ANOSIM test was performed using α = 0.05 for statistical significance. Average similarity within groups was calculated using SIMPER (Clarke, 1993). Diversity was measured using Shannon-Weaver diversity index. These analyses were performed using Primer 6 (Clarke and Gorley, 2006). To estimate depth of sequence sampling, rarefaction curves were obtained for each sample using PAST 3.03 (Hammer et al., 2001) and EstimateS 9.1.0 (Colwell, 2005). For mean comparisons two-way ANOVA test was performed using two-tailed *p*-values and α = 0.05. Linear discriminant analysis (LDA) effect size tool-LEfSe (Segata et al., 2011) was used to identify Operational Taxonomical Units (OTUs) with differential relative abundance comparing healthy and dandruff subject samples from each body site. For Kruskal-Wallis test, α = 0.05 was used as a cut-off for statistical significance. LDA score was calculated for OTUs with *p* ≤ 0.05 (LDA score threshold ≥2.00). Differentially abundant OTUs were used to calculate the microbial dysbiosis index (MD-index), defined as the log of (total abundance of OTUs increased in dandruff) over (total abundance of OTUs decreased in dandruff) (Gevers et al., 2014).

## Results

### Data collection and sequence analysis

We analyzed bacterial and fungal communities in 48 skin samples from 11 healthy and 13 dandruff individuals. Scalp and forehead samples were studied to assess variation across two sebaceous body areas and to enable comparison between lesional and non-lesional skin sites in dandruff subjects.

*In silico* analyses were performed to select primer sets for bacteria and fungi considering specificity and coverage. Amplicons containing the V4 hypervariable region of 16S rRNA gene from bacteria and the ITS1 region from fungi were sequenced using Illumina MiSeq platform.

We obtained ~2.3 million reads for bacteria and 1.4 million for fungi after quality and size filtering (Supplementary Table 2). Approximately 88% of the bacterial sequences were assigned using Greengenes database, and 612 Operational Taxonomic Units (OTUs) were found. Chloroplast and mitochondria sequences (nearly 8% of the reads) were removed from the analysis. For fungi, only approximately half of the sequences (51.4%) were assigned using UNITE database. Therefore, we performed a second step of analysis consisting of sequence clustering and assignment using GenBank to allow a larger and more reliable panorama of the fungal communities. This strategy increased the percentage of assigned sequences (>83%). A total of 274 fungal OTUs were found. Moreover, the method allowed the detection of contaminant sequences assigned to non-fungal taxonomic groups (7.8% of the reads). They correspond to plants and were removed from the analyses. Rarefaction curves were generated to estimate depth of sequence sampling (Supplementary Figures 1, 2).

### Taxonomic composition of microbial communities

**Figure 1:**
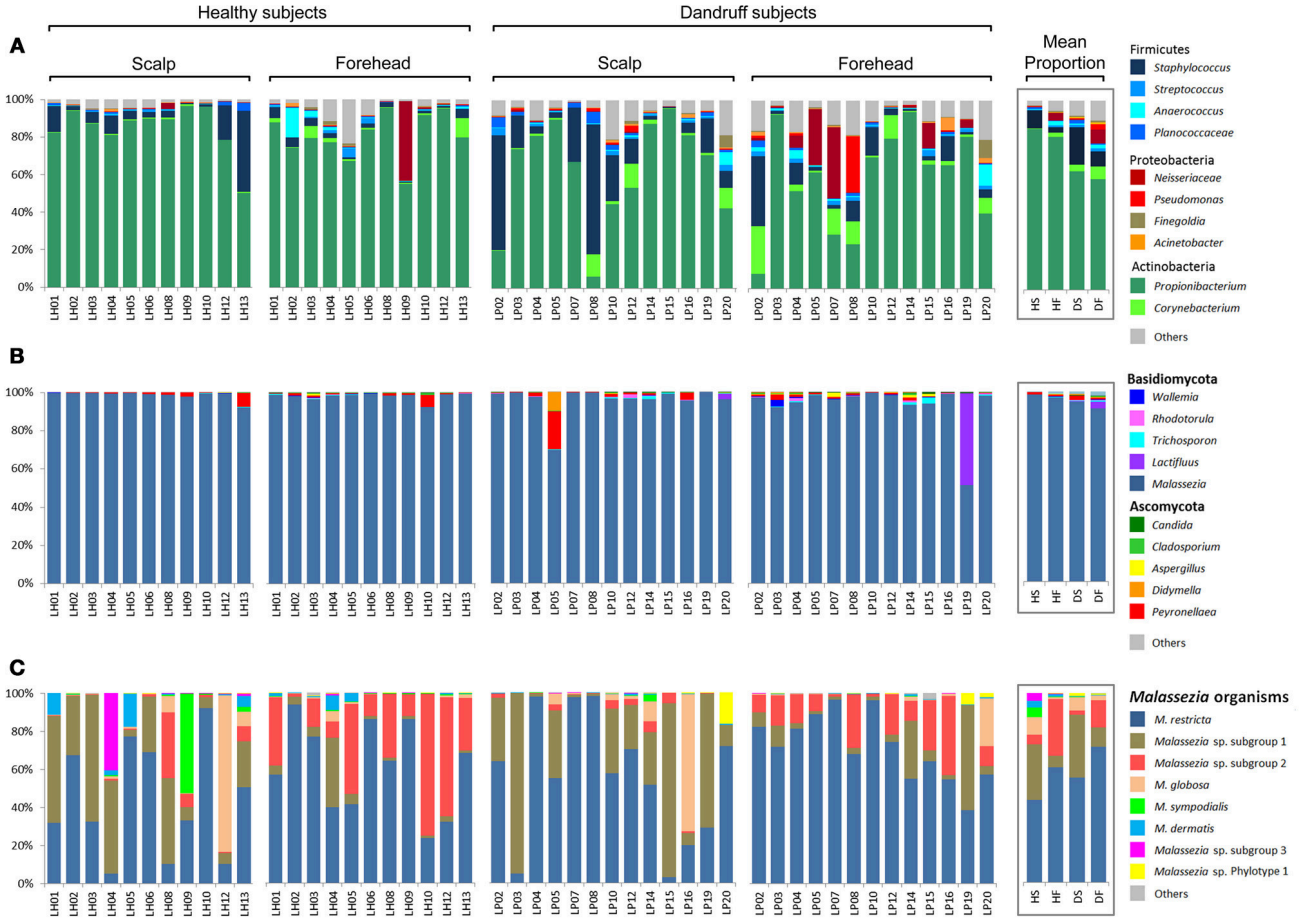
**Skin microbial composition in healthy and dandruff subjects**. Relative abundance of **(A)** Bacteria at genus level; **(B)** Fungi at genus level; and **(C)** *Malassezia* at species level. Mean proportions according to health status and body site are shown in the box. HS, scalp samples from healthy subjects; HF, forehead samples from healthy subjects; DS, scalp samples from dandruff subjects; DF, forehead samples from dandruff subjects.

*Actinobacteria, Firmicutes*, and *Proteobacteria* were the most abundant bacterial phyla. At genus level, *Propionibacterium, Staphylococcus*, and *Corynebacterium* were found to be the three most abundant genera in both healthy and dandruff subjects (Figure 1A). *Malassezia* sp. comprised the vast majority of fungi in almost all of the samples (~96% of the reads; Figure 1B); therefore, we also performed taxonomic assignment at *Malassezia* species level. Nine *Malassezia* formally described species were detected (*M. restricta, M. globosa, M. sympodialis, M. dermatis, M. japonica, M. obtusa, M. pachydermatis, M. sloofiae*, and *M. furfur*), as well as an uncharacterized phylotype previously described (LCP-2008a—Genbank accession number EU192362). *M. restricta* was the most abundant species. After the 2-step OTU assignment for fungi, other uncharacterized *Malassezia* organisms were found, corresponding to more than 37% of the reads. Their sequences are dissimilar from all *Malassezia* type strains, thus were not assigned to any formally described species. The uncharacterized *Malassezia* sequences formed three subgroups (≥95% sequence similarity), two of them being highly abundant: *Malassezia* sp. subgroup 1 was found in all 48 samples, comprising 26.72% of the sequences; and subgroup 2 was found in 46 samples (9.6% of the sequences; Figure 1C).

### Microbial communities in health and dandruff

**Figure 2:**
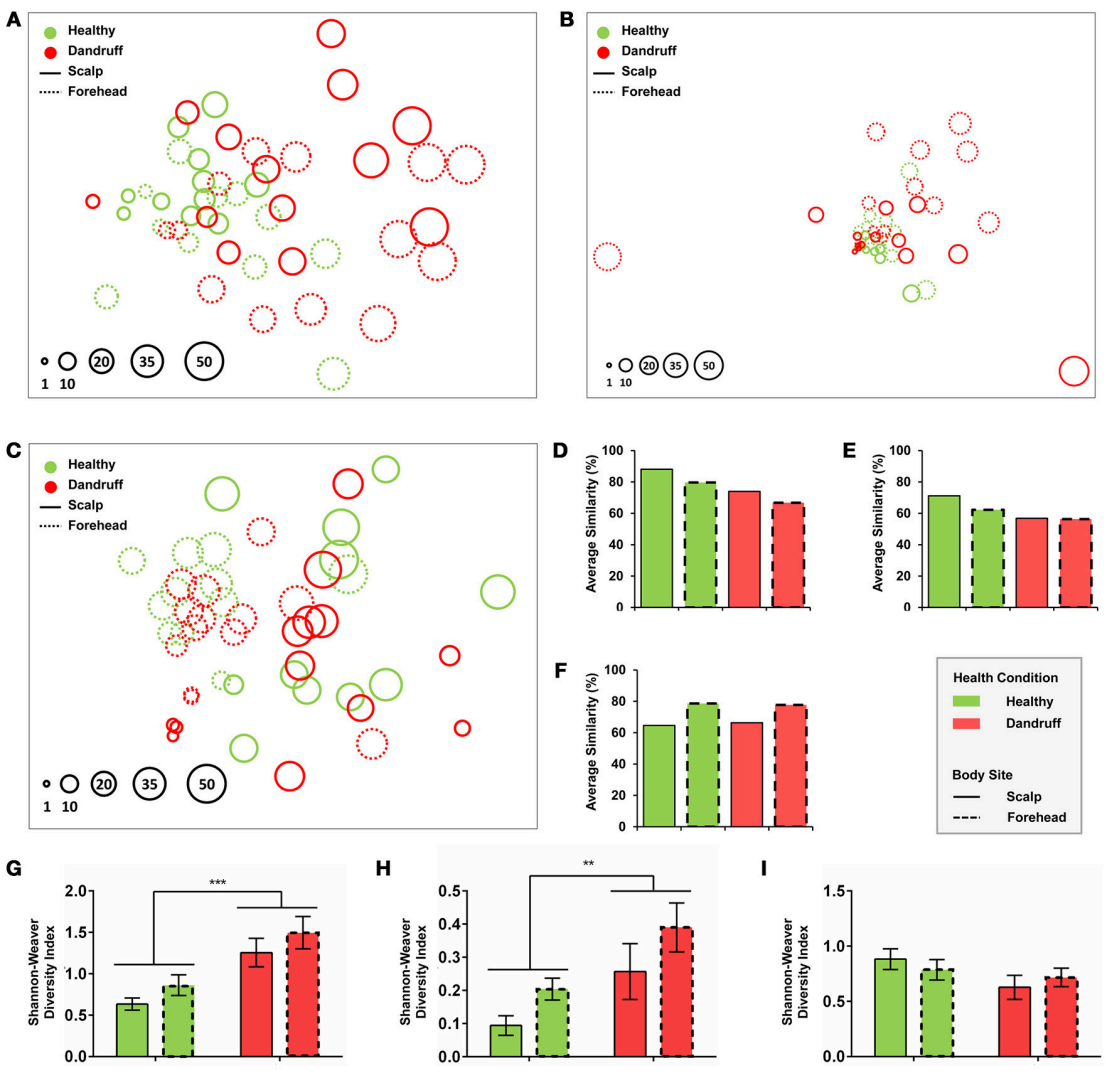
**Community and diversity analyses**. Non-Metrical Multidimensional Scaling (nmMDS) of **(A)** Bacteria at genus level, **(B)** Fungi at genus level, and **(C)** *Malassezia* at species level showing 48 samples from healthy and dandruff subjects. Each circle represents one sample, and diameters are proportional to the Shannon-Weaver diversity (lowest value indexed as 1). Average similarity (SIMPER) for **(D)** Bacteria at genus level, **(E)** Fungi at genus level and **(F)** *Malassezia* at species level. Mean diversity based on Shannon-Weaver Index for **(G)** Bacteria at genus level, **(H)** Fungi at genus level, and **(I)** *Malassezia* at species level. Significance was determined by Two-way ANOVA Test. Bars represent Mean ± SEM. **p < 0.005; ***p < 0.001.

Statistical analyses using non-metrical Multidimensional Scaling and ANOSIM revealed that healthy bacterial and fungal communities at genus level clustered according to body site, indicating that scalp and forehead from healthy subjects harbor distinct microbiotas (bacteria: *p* = 0.023, *R* = 0.122; fungi: *p* = 0.005, *R* = 0.188; Figures 2A,B). Within each body site group, scalp samples had higher intra-group similarity than forehead samples, as revealed by similarity percentage (SIMPER analysis) (Figures 2D,E).

Bacterial and fungal communities at genus level were more dissimilar among samples from dandruff subjects as compared with healthy subjects (Figures 2A,B,D,E). Despite the clinical differences between samples from scalp (lesional site) and forehead (non-lesional site) from dandruff subjects, the variations between the sites resembles the differences observed between scalp and forehead from healthy subjects, indicating that dandruff condition is related to dysbiosis in both clinically involved and uninvolved skin sites. Analysis using Shannon-Weaver index revealed that diversity was significantly higher in dandruff samples than in healthy ones for both bacteria (*p* = 0.0002; Figure 2G) and fungi (*p* = 0.0095; Figure 2H) at genus level. Diversity showed no correlation with number of reads from each sample from both bacteria (*p* = 0.4284) and fungi (*p* = 0.7219).

*Malassezia* microbiota at species level also differed between body sites in healthy subjects (*p* = 0.001, *R* = 0.393; Figure 2C). However, contrasting with genus level analyses, in dandruff subjects the average similarity among samples did not differ in relation to healthy subjects for both scalp and forehead samples (Figure 2F). Shannon-Weaver diversity indexes obtained for healthy and dandruff subjects were not statistically different either (Figure 2I), suggesting that the diversity of *Malassezia* community at species level was not associated with dandruff condition.

### Differential abundance of bacterial and fungal taxa

**Figure 3:**
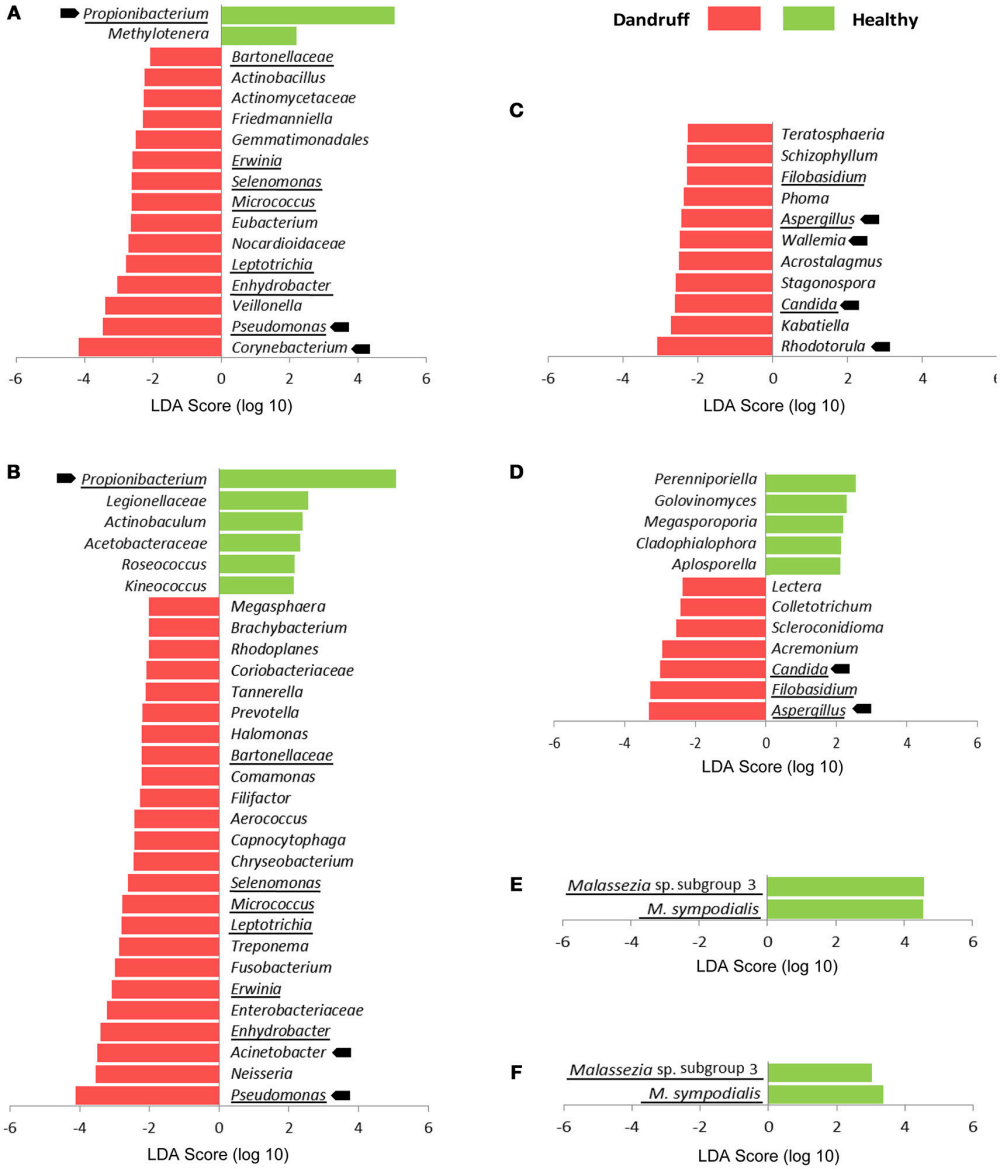
**Differentially abundant OTUs stratified by body site and health status. (A)** Bacteria at genus level from scalp samples; **(B)** Bacteria at genus level from forehead samples; **(C)** Fungi at genus level from scalp samples; **(D)** Fungi at genus level from forehead samples. **(E)** *Malassezia* at species level from scalp samples; **(F)** *Malassezia* at species level from forehead samples. Abundant OTUs are indicated by arrows; OTUs differentially abundant in both skin sites are underlined. LDA score threshold was set to ≥2.0. Kruskal-Wallis *p* < 0.05 was considered statistically significant.

We also searched for specific OTUs that could be differentially abundant between groups using Linear discriminant analysis effect size tool (LEfSe). Considering bacteria and fungi at genus level, remarkably more OTUs were found to be overrepresented in scalp and forehead samples from dandruff subjects in comparison with healthy ones (Figures 3A–D). These findings corroborate the occurrence of dysbiosis in lesional as well as non-lesional body sites. Bacterial genera *Pseudomonas, Leptotrichia, Micrococcus, Selenomonas, Erwinia, Enhydrobacter*, and *Bartonellaceae* were significantly more abundant in dandruff subject samples in both skin sites, whereas *Propionibacterium* was underrepresented (Figures 3A,B). In the case of fungi, dandruff samples from both sites were enriched in genera *Candida, Aspergillus*, and *Filobasidium* (Figures 3C,D). Analysis of *Malassezia* microbiota at species level did not reveal OTUs overrepresented in dandruff subjects, either in scalp or forehead samples. Two OTUs of low prevalence were found to be underrepresented in dandruff, one was an uncharacterized *Malassezia* subgroup (Figures 3E,F).

**Figure 4:**
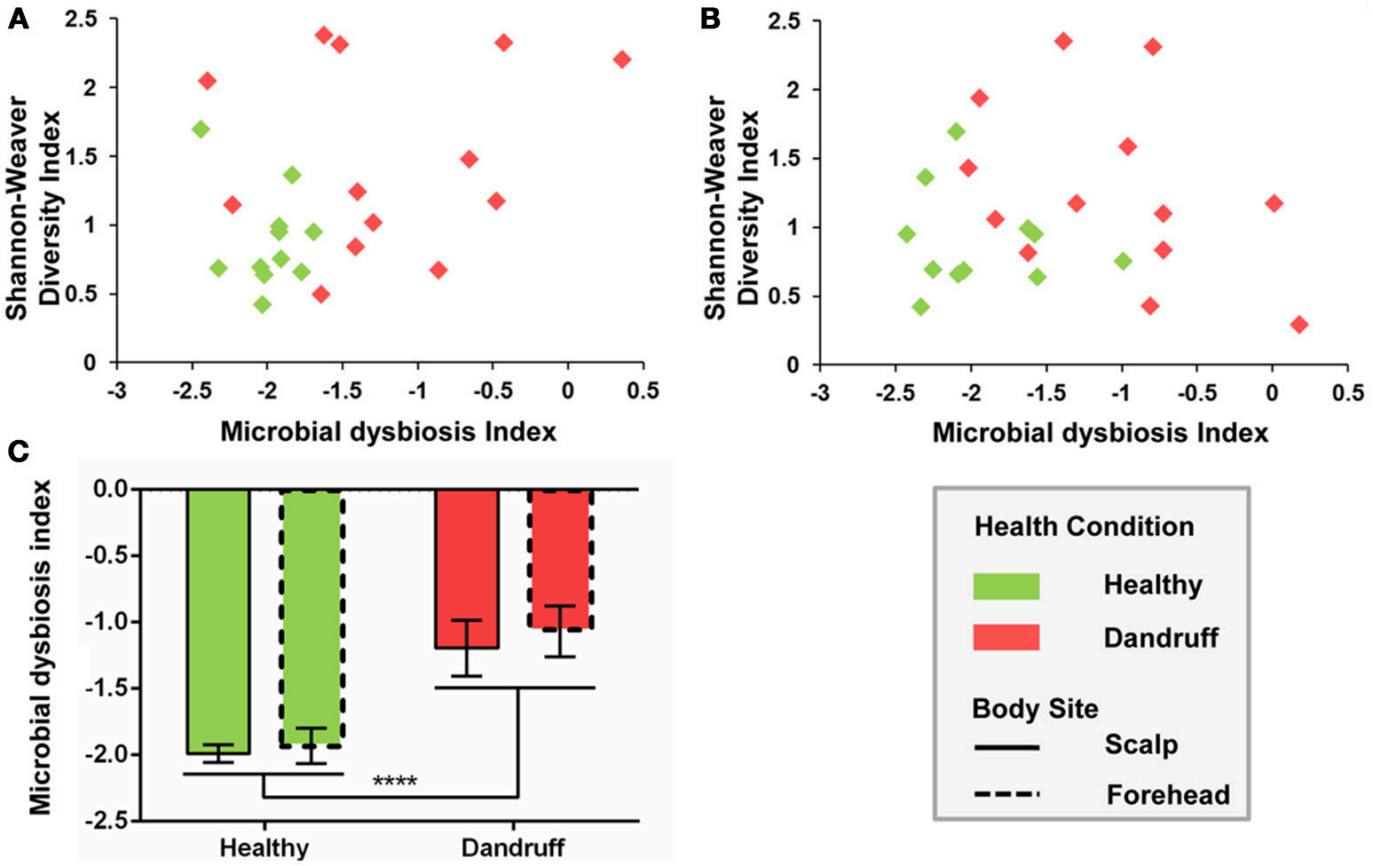
**Microbial dysbiosis index (MD-index) for bacterial communities according to health status. (A)** Scatter plot of MD-index vs. Shannon-Weaver diversity for scalp samples; **(B)** Scatter plot of MD-index vs. Shannon-Weaver diversity for forehead samples; **(C)** Average MD-index. Significance was determined by Two-way ANOVA Test. Bars represent Mean ± SEM. ****p < 0.0001.

Microbial dysbiosis index (MD-index), based on differentially abundant bacterial OTUs, differed according to health status. Samples from healthy subjects showed overall lower MD-indexes as well as lower diversity for both scalp and forehead, when compared with samples from dandruff subjects (Figures 4A,B). Average MD-index was significantly higher in dandruff samples than in healthy ones (*p* < 0.0001; Figure 4C). MD-index could not be calculated for fungi because there were no underrepresented OTUs in scalp samples; and in the case of forehead, some samples lacked some of the underrepresented OTUs.

## Discussion

Microbial communities inhabiting the human body and their relation with diseases have been the subject of many studies and intense team efforts (The Human Microbiome Project Consortium, 2012a,b; The Integrative HMP (iHMP) Research Network Consortium, 2014). In the case of dermatopathologies, the association with microbiome imbalances is often not well-established. Many skin diseases and disorders have been primarily associated with one specific group of microorganisms, e.g., acne vulgaris (Das and Reynolds, 2014), rosacea (Casas et al., 2012), seborrheic dermatitis, and dandruff (Hay, 2011); although the possible microbial role in the development of symptoms is not completely understood.

Despite its high prevalence, the shortage of effective treatments, and the impact of symptoms (Piérard-Franchimont et al., 2006a), the etiology of dandruff remains poorly understood. Knowledge regarding seborrheic dermatitis, a related condition that has been often considered a more severe form of dandruff affecting body areas other than the scalp (Piérard-Franchimont et al., 2006a; Schwartz et al., 2013), is also limited. The association of these conditions with *Malassezia* yeasts has been reported (Gemmer et al., 2002; DeAngelis et al., 2005; Tajima et al., 2008; Clavaud et al., 2013); however, it is still unclear whether they are actually the causal agent of dandruff, and if there are other microorganisms involved in the pathogenic process.

**Figure 5:**
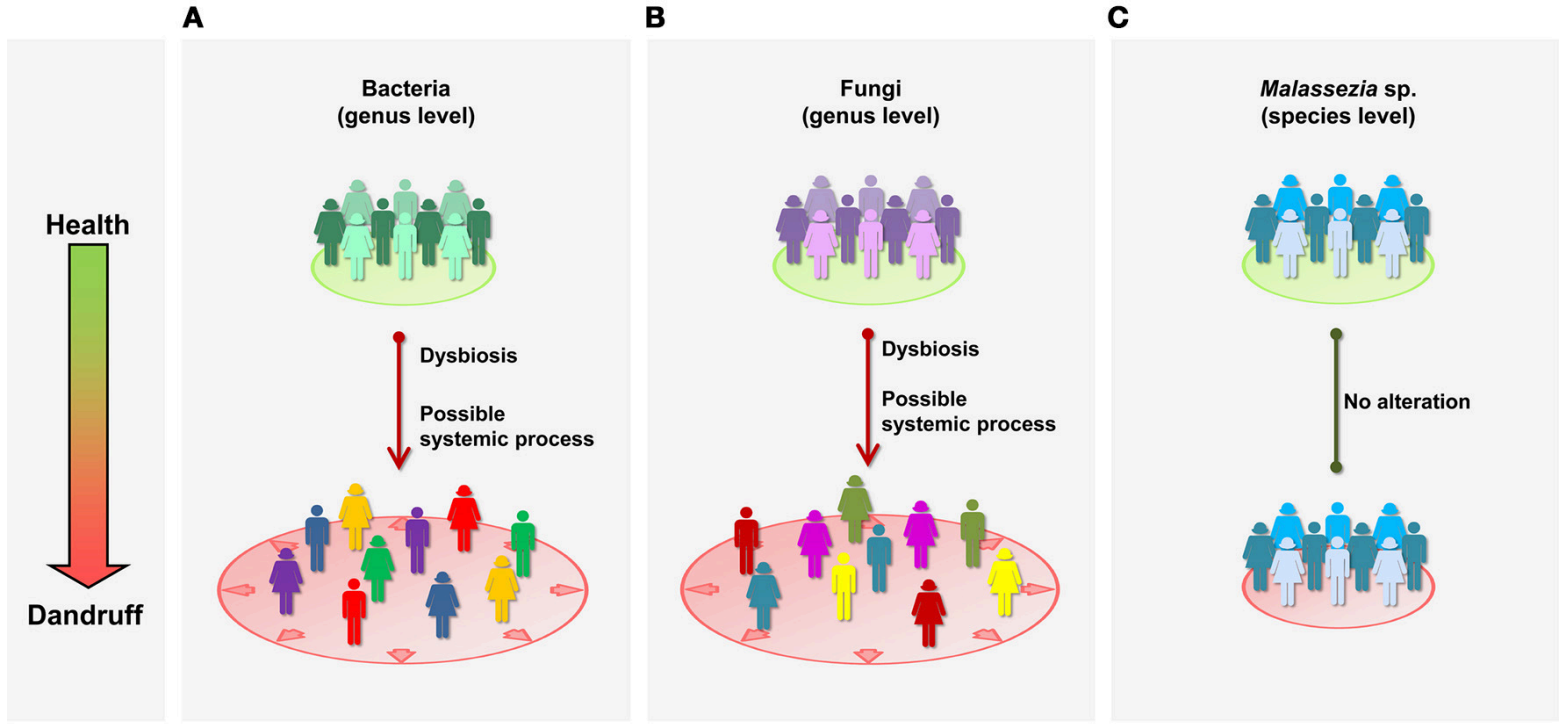
**Schematic representation of microbial communities in health and dandruff. (A)** Bacteria at genus level; **(B)** Fungi at genus level; and **(C)** *Malassezia* at species level. Different colors represent distinct communities; greater distance between figures represent less intra-group similarity. “Systemic” refers to general processes in the body, as opposed to specific local sites.

Here, we report the characterization of bacterial and fungal microbiome from healthy and dandruff subjects. Lesional and non-lesional skin sites were examined, revealing that bacterial and fungal communities at genus level are imbalanced in dandruff subjects, even in non-lesional sites (Figures 5A,B). These findings suggest that the process associated with dandruff may be systemic, and not restricted to the skin site exhibiting the symptoms. An instability of the skin microenvironment at systemic level, possibly due to immune response alterations, might lead to dysbiosis, disruption of skin barrier (Gallo and Nakatsuji, 2011; Hooper et al., 2012) and to clinical manifestation of dandruff in specific skin sites. This could have implications for therapeutic approaches: since dandruff manifests locally, treatment is normally not systemic, which may explain difficulties in controlling symptoms. In the future, studies of microbial communities from a greater number of body sites should be performed to further support the hypothesis and to evaluate the extent of the alterations in the body. Dysbiosis has been associated with various immune-related conditions; however, most cases involve loss of microbial diversity, as consequence of the expansion of potentially harmful microorganisms or loss of beneficial species (Petersen and Round, 2014). Specifically regarding skin disorders, a loss of bacterial diversity has been reported in patients with atopic dermatitis (Kong et al., 2012) and psoriasis (Alekseyenko et al., 2013), in comparison with healthy subjects. Our data suggest that a different phenomenon occurs in dandruff, as microbial diversity was higher than in healthy subjects. The skin environment in dandruff might become less selective for microbial growth, which could also account for the decrease of intragroup similarly of dandruff bacterial and fungal communities.

Although fungal community is dominated by *Malassezia* genus, consistent with previous studies of healthy skin (Findley et al., 2013; Oh et al., 2014), we found no evidence of association between *Malassezia* and dandruff. Moreover, in contrast with microbial shifts observed at genus level, *Malassezia* microbiota at species level was not altered in dandruff, suggesting it is not affected by possible systemic alterations taking place in dandruff subjects (Figure 5C). It is surprising that *Malassezia* communities from scalp samples were similar in diversity and composition to one another regardless of whether they are from healthy or dandruff subjects.

Bacterial and fungal microbiome associated with dandruff has been previously studied in scalp samples (lesional site) through cloning and Sanger sequencing, showing that the ratios between abundant bacterial and fungal organisms shifted in dandruff compared with healthy subjects, especially *Malassezia restricta, Propionibacterium* sp., and *Staphylococcus* sp. (Clavaud et al., 2013; Wang et al., 2015). More recently, Xu et al. (2016) showed that host factors such as skin sebum and water content are related to dandruff. Analyzing scalp microbiome by NGS, the authors found that dandruff is more closely related to bacteria than to fungi, and reported no significant association with *Malassezia* at species level, although some OTUs showed positive or negative correlation with dandruff (Xu et al., 2016). Our results corroborate the relationship between dandruff and bacteria but not with *Malassezia* at species level, and showed for the first time increased diversity and dissimilarity among dandruff samples, affecting also asymptomatic skin sites.

A bioinformatics strategy based on sequence clustering and OTU assignment using combined databases increased the percentage of fungal assigned sequences, and revealed the presence of uncharacterized *Malassezia* organisms. Previously, we have reported *Malassezia* sequences that were dissimilar from any described species in healthy, psoriasis and seborrheic dermatitis subjects (Paulino et al., 2006, 2008; Soares et al., 2015). A more comprehensive analysis shown here emphasized the importance of such uncharacterized organisms, found in large proportions in most samples regardless of the health status. Such findings highlight the significance of developing strategies to assess unassigned DNA sequences, in order to ensure a reliable view of fungal communities. Further, studies are needed, including the isolation and complete characterization of these organisms, as well as phylogenetic analysis using DNA regions other than ITS1, which contains large insertions and deletions that restrict the reliability of the sequence alignment.

Data presented here indicate that dandruff is associated with bacterial and fungal dysbiosis and suggests a systemic process affecting even asymptomatic skin sites. Findings showed that microbiome analyses, particularly of fungal communities, require strategies to assess unclassified sequences, otherwise useful information might be lost. Further, studies of larger cohorts and more extensive topographical analysis, immune response, functional characterization of microbial communities, and bacterial-fungal interactions might generate novel hypotheses and provide new perspectives on the pathogenesis of dandruff and other skin diseases, as well as to expand our understanding of healthy skin microbiota.

## Author contributions

LP, CC, and LB conceived the study; RS, VD, PC, and AB performed the experiments; RS, PC, AB, and LP analyzed the data; RS, PC, RD, CC, LB, and LP interpreted and discussed the results; RS and LP wrote the paper.

### Conflict of interest statement

The authors declare that the research was conducted in the absence of any commercial or financial relationships that could be construed as a potential conflict of interest.
