# Supplementary material for: Dysbiotic Bacterial and Fungal Communities Not Restricted to Clinically Affected Skin Sites in Dandruff
Source: Front Cell Infect Microbiol. 2016 Nov 17;6:157. doi: 10.3389/fcimb.2016.00157 (PMC5112237; doi:10.3389/fcimb.2016.00157)
Supplement: Supplementary file 1 [file Presentation1.PDF]

Supplementary Material

Dysbiotic bacterial and fungal communities not restricted to clinically affected skin sites in dandruff

Soares, RC[1]; Camargo-Penna, PH[1]; de Moraes, VCS[1]; De Vecchi, R[2]; Clavaud, C[3]; Breton, L[3]; Braz, ASK[1]; Paulino, LC[1]*

*** Correspondence:**

Luciana C. Paulino

luciana.paulino@ufabc.edu.br

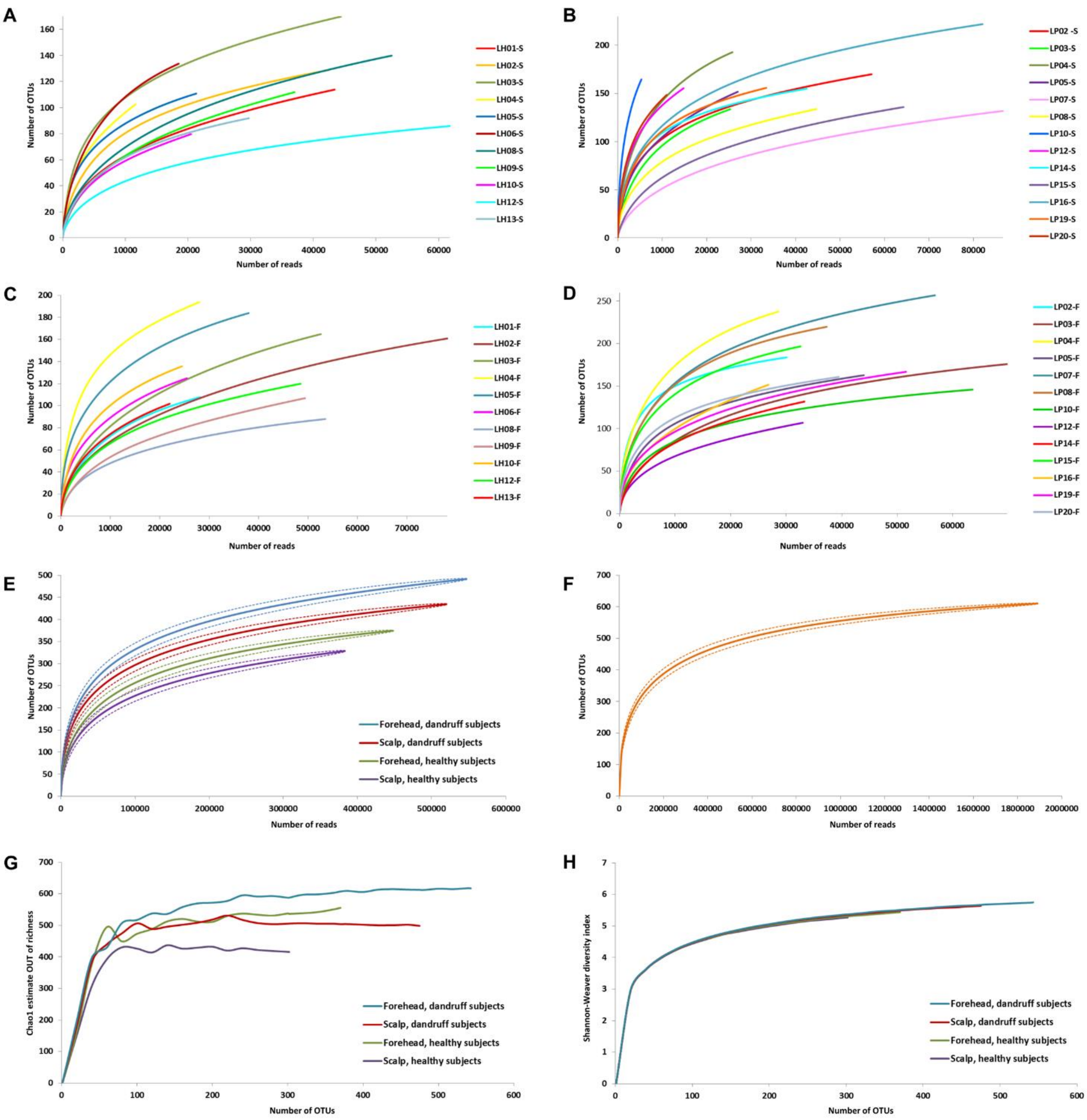

Supplementary Figure 1: Rarefaction plots for bacterial communities. (**A**) Scalp samples from healthy subjects; (**B**) Scalp samples from dandruff subjects; (**C**) Forehead samples from healthy subjects and (**D**) Forehead samples from healthy subjects; (**E**) Total per health status and body site (dotted lines represent the 95% CI); (**F**) Overall total (dotted lines represent the 95% CI); (**G**) Chao1 richness; (**H**) Shannon-Weaver diversity index. Chao 1 and Shannon-Weaver index were calculated using log of [number of reads].

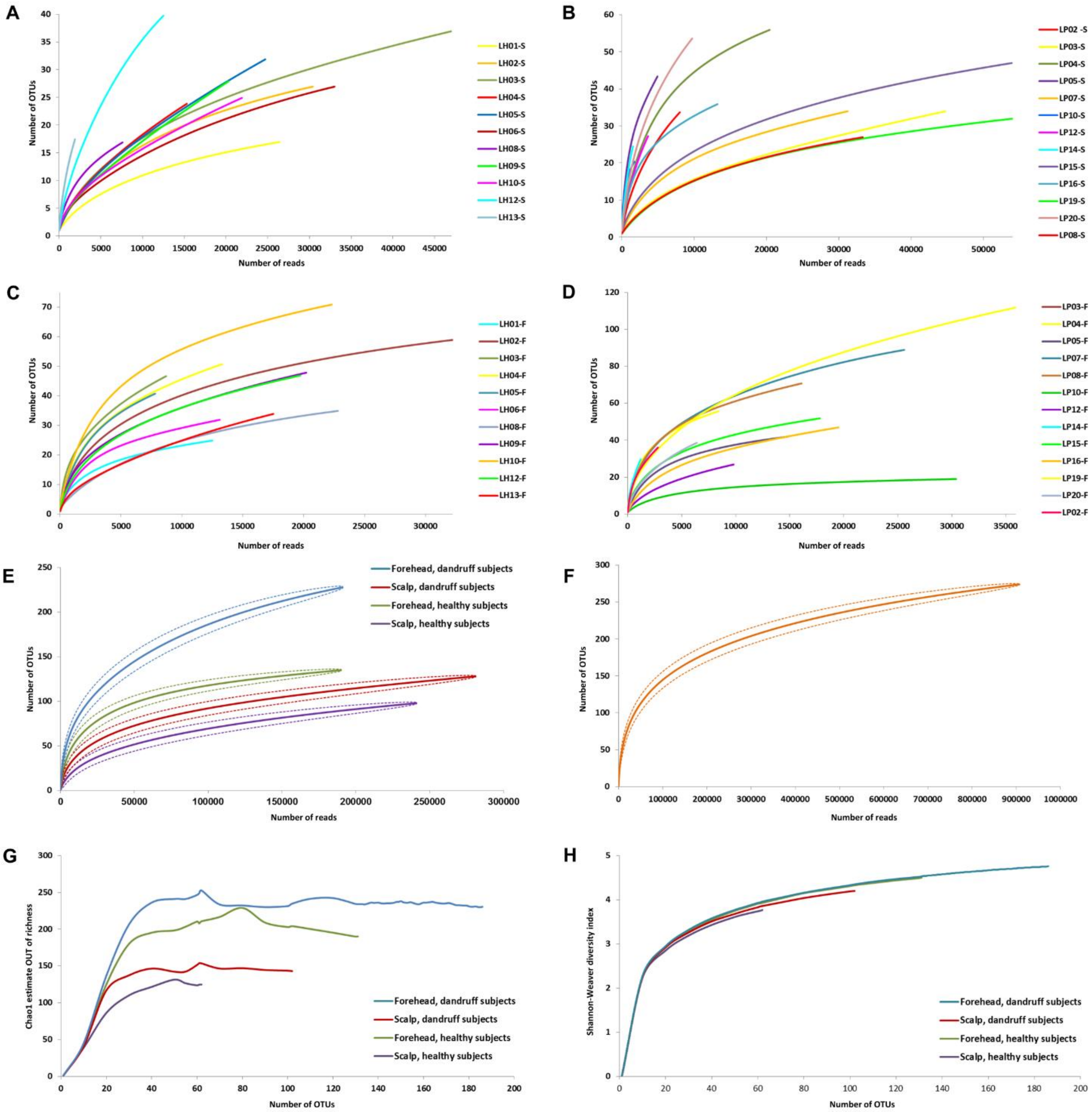

Supplementary Figure 2: **Rarefaction plots for fungal communities.** (**A**) scalp samples from healthy subjects; (**B**) scalp samples from dandruff subjects; (**C**) forehead samples from healthy subjects and (**D**) forehead samples from healthy subjects; (**E**) Total per health status and body site (dotted lines represent the 95% CI); (**F**) Overall total (dotted lines represent the 95% CI); (**G**) Chao1 richness; (**H**) Shannon-Weaver diversity index. Chao 1 and Shannon-Weaver index were calculated using log of [number of reads].

Supplementary Table 1: Subjects included in the study.

Subject	Age	Gender	Health Status	Dandruff Score
LH01	57	F	Healthy	-----
LH02	35	M	Healthy	-----
LH03	61	M	Healthy	-----
LH04	57	F	Healthy	-----
LH05	52	F	Healthy	-----
LH06	52	M	Healthy	-----
LH08	36	F	Healthy	-----
LH09	25	F	Healthy	-----
LH10	36	F	Healthy	-----
LH12	57	F	Healthy	-----
LH13	18	M	Healthy	-----
LP02	45	F	Dandruff	2.06
LP03	25	F	Dandruff	6.12
LP05	25	M	Dandruff	0.25
LP04	30	F	Dandruff	ND
LP07	31	F	Dandruff	0.25
LP08	28	F	Dandruff	2.31
LP10	30	F	Dandruff	4.18
LP12	42	M	Dandruff	5.06
LP14	46	M	Dandruff	4.93
LP15	46	F	Dandruff	0.68
LP16	46	M	Dandruff	3.81
LP19	34	M	Dandruff	5.37
LP20	39	M	Dandruff	5.37

F: female, M: male; ND: Not determined.

Supplementary Table 2: Bacterial and fungal sequence data.

Sample	Bacteria					Fungi				
	Number of Reads	Assigned sequences (%)	Bacterial sequences (%)	Non bacterial sequences (%)	Not assigned sequences (%)	Number of Reads	Assigned sequences - UNITE (%)	Assigned sequences - UNITE + GENBANK (%)	Nonfungal sequences (%)	Not assigned sequences (%)
LH01-S	48341	90.40	90.14	0.26	9.60	30330	40.85	90.29	0.33	9.38
LH02-S	44789	95.56	95.21	0.36	4.44	40167	53.64	77.69	0.33	21.98
LH03-S	49507	90.57	89.95	0.62	9.43	54636	30.17	87.83	0.45	11.72
LH04-S	13674	87.13	85.79	1.34	12.87	37586	17.38	54.12	0.06	45.83
LH05-S	24335	88.95	88.20	0.74	11.05	41004	64.58	67.00	0.22	32.78
LH06-S	21274	88.58	87.62	0.96	11.42	38662	67.36	93.17	0.04	6.79
LH08-S	56831	92.67	92.63	0.04	7.33	11151	31.75	86.40	0.19	13.42
LH09-S	39826	93.25	93.18	0.08	6.75	22366	83.69	96.14	0.05	3.81
LH10-S	23474	88.02	87.97	0.05	11.98	24844	88.40	94.54	0.11	5.35
LH12-S	66437	93.32	93.30	0.01	6.68	32191	62.99	65.66	0.50	33.84
LH13-S	33369	89.34	89.31	0.04	10.66	3260	53.99	72.76	0.34	26.90
LH01-F	31257	90.90	90.53	0.37	9.10	23186	35.84	57.59	0.47	41.94
LH02-F	88362	89.89	88.71	1.18	10.11	34869	89.66	95.00	1.36	3.64
LH03-F	58489	90.42	90.19	0.23	9.58	35702	92.18	96.84	0.27	2.89
LH04-F	36675	86.11	76.64	9.47	13.89	39986	27.67	42.82	17.52	39.65
LH05-F	116594	85.49	32.68	52.81	14.51	68609	6.38	12.36	70.44	17.21
LH06-F	28396	90.26	90.19	0.07	9.74	22666	87.98	95.62	0.04	4.34
LH08-F	56737	94.64	94.59	0.05	5.36	31624	49.72	74.85	0.04	25.11
LH09-F	56644	87.90	87.50	0.40	12.10	26975	85.69	95.15	0.37	4.47
LH10-F	27807	88.88	88.66	0.22	11.12	37053	22.24	64.05	0.02	35.93
LH12-F	52326	93.00	92.89	0.11	7.00	23846	33.81	86.84	0.85	12.31
LH13-F	26525	84.32	83.60	0.72	15.68	21489	62.48	86.03	0.54	13.43
LP02-S	66074	86.89	86.62	0.27	13.11	10000	59.00	87.37	0.02	12.61
LP03-S	28717	89.15	88.75	0.40	10.85	53326	5.10	84.65	0.01	15.34
LP04-S	30506	85.90	84.91	0.99	14.10	22171	94.36	95.99	0.05	3.96
LP05-S	31034	87.91	87.44	0.47	12.09	7771	57.11	73.63	0.15	26.21
LP07-S	95890	90.71	90.60	0.11	9.29	33012	95.87	97.46	0.06	2.48
LP08-S	51081	88.05	87.75	0.30	11.95	34969	95.92	97.37	0.01	2.63
LP10-S	7717	73.86	70.56	3.30	26.14	3138	43.24	66.41	2.49	31.10
LP12-S	18064	84.36	82.98	1.38	15.64	5050	59.33	78.30	1.70	20.00
LP14-S	47498	91.34	89.74	1.60	8.66	2864	49.09	67.70	3.91	28.39
LP15-S	67390	96.07	95.70	0.37	3.93	64640	4.40	84.53	0.32	15.15
LP16-S	92748	89.28	88.68	0.60	10.72	29126	86.65	89.79	1.59	8.62
LP19-S	38921	87.38	86.25	1.13	12.62	75343	21.82	72.42	0.09	27.49
LP20-S	15883	71.60	69.99	1.61	28.40	17184	90.12	96.20	0.00	3.80
LP02-F	37690	80.75	80.32	0.43	19.25	8753	86.06	91.55	0.01	8.44
LP03-F	74725	93.87	93.62	0.25	6.13	2304	57.07	72.92	0.09	27.00
LP04-F	37794	80.22	76.00	4.23	19.78	30775	89.61	94.08	0.34	5.58
LP05-F	48835	90.56	90.37	0.18	9.44	21612	88.19	95.30	0.01	4.69
LP07-F	73234	79.47	77.70	1.77	20.53	28384	90.34	92.77	0.49	6.74
LP08-F	51066	80.54	73.33	7.21	19.46	31686	51.99	67.40	0.60	32.00
LP10-F	73338	87.42	86.93	0.49	12.58	32128	93.10	96.47	0.24	3.28
LP12-F	37411	88.98	88.52	0.46	11.02	11720	65.67	86.34	0.81	12.85
LP14-F	139376	87.37	23.99	63.38	12.63	65542	1.69	2.47	77.44	20.09
LP15-F	38701	85.13	84.66	0.47	14.87	21840	64.26	88.79	0.31	10.90
LP16-F	31306	87.28	86.21	1.07	12.72	23594	54.39	90.19	0.59	9.22
LP19-F	59593	88.74	86.87	1.86	11.26	43809	67.37	89.73	0.00	10.27
LP20-F	50662	78.66	78.36	0.30	21.34	16276	57.63	63.50	0.50	36.00
**TOTAL**	**2346923**	**88.42**	**81.20**	**7.23**	**11.58**	**1399219**	**51.40**	**75.48**	**7.86**	**16.66**

Sample code: -S: scalp; -F: forehead
